# Measurement of Shared Social Identity in Singing Groups for People With Aphasia

**DOI:** 10.3389/fpsyg.2021.669899

**Published:** 2021-06-17

**Authors:** Mark Tarrant, Ruth A. Lamont, Mary Carter, Sarah G. Dean, Sophie Spicer, Amy Sanders, Raff Calitri

**Affiliations:** ^1^College of Medicine and Health, University of Exeter, Exeter, United Kingdom; ^2^Department of Pharmacy and Pharmacology, University of Bath, Bath, United Kingdom; ^3^College of Medical and Dental Sciences, University of Birmingham, Birmingham, United Kingdom

**Keywords:** social identity, stroke, cohesiveness, groups, process evaluation, health, aphasia, singing

## Abstract

Community groups are commonly used as a mode of delivery of interventions for promoting health and well-being. Research has demonstrated that developing a sense of shared social identity with other group members is a key mechanism through which the health benefits of group membership are realized. However, there is little understanding of how shared social identity emerges within these therapeutic settings. Understanding the emergence of shared social identity may help researchers optimize interventions and improve health outcomes. Group-based singing activities encourage coordination and a shared experience, and are a potential platform for the development of shared social identity. We use the “*Singing for People with Aphasia*” (SPA) group intervention to explore whether group cohesiveness, as a behavioral proxy for shared social identity, can be observed and tracked across the intervention. Video recordings of group sessions from three separate programmes were rated according to the degree of cohesiveness exhibited by the group. For all treatment groups, the final group session evidenced reliably higher levels of cohesiveness than the first session (*t* values ranged from 4.27 to 7.07; all *p* values < 0.003). As well as providing confidence in the design and fidelity of this group-based singing intervention in terms of its capacity to build shared social identity, this evaluation highlighted the value of observational methods for the analysis of shared social identity in the context of group-based singing interventions.

## Introduction

Group singing, and the arts more generally, is often used in community settings to promote and maintain health and well-being (World Health Organisation, 2019). Evidence supports the effectiveness of these interventions among a variety of groups and in a number of health domains (Poscia et al., 2018; World Health Organisation, 2019; Pohl et al., 2020). A recent World Health Organization review of arts and music-based interventions commented on the role of the group context alongside arts-based activity in allowing for experiences of social cohesion and meaningful connection to others (World Health Organisation, 2019). More formally, group interventions that promote the development of *shared social identity (SSI)* among group members appear more likely to elicit the intended outcomes (e.g., improved health and well-being) than those interventions in which recipients fail to come together *as a group*, or are characterized by high levels of conflict between group members (Sani, 2005; Nackers et al., 2015; Borek et al., 2019; Tarrant et al., 2020). By understanding the social identity processes that contribute to positive (health) outcomes, and the factors that account for variability in their success, group-based singing (and other group) interventions can be optimized (Moore et al., 2015; Richards and Hallberg, 2015; Levati et al., 2016). To this end, the current paper reports on the emergence and progression of SSI in the context of a group-based singing intervention for people with post-stroke aphasia (Tarrant et al., 2018).

## Shared Social Identity in Group-Based Interventions

A substantial body of evidence shows that shared social identity (SSI)—the sense of connection and meaning that individuals may develop in group-based interventions—is an important determinant of health outcomes (Haslam et al., 2009, 2018). Summarizing this evidence, a recent systematic review and meta-analysis of 27 studies that sought to build SSI, found a moderate-to-strong impact of the group interventions on health outcomes, and strongest effects emerging in interventions that successfully built SSI (Steffens et al., 2019). Haslam et al. (2018) have articulated the core hypotheses that specify relationships between group membership, SSI processes, and health. A key hypothesis from the *Social Identity Approach to Health* is the identification hypothesis, which states that *“a person will generally experience the health-related benefits or costs of a given group membership only to the extent that they identify with that group”* (p. 17). Among other things, this means that when someone identifies themselves in terms of a social group membership (e.g., as a member of an intervention group), the group has a greater potential to facilitate access to the range of psychological resources that promote health and well-being.

Indeed, group-based interventions that equip people with the necessary knowledge, skills and confidence to form new social identities have been shown to have a marked impact on psychosocial health (e.g., reduced loneliness, depression and anxiety, and improved well-being among adults) (Haslam et al., 2016, 2019). Explaining the process underpinning these effects, research has shown that identifying with a social group provides the foundation for the provision and receipt of essential social support, acts as a buffer to protect against negative social stigma, provides meaning and connection, allows for positive social influence, and nurtures a sense of collective efficacy (Levine and Thompson, 2004; Smith and Woodworth, 2012; Alnabulsi and Drury, 2014; Greenaway et al., 2016; Tarrant et al., 2017, 2020; Haslam et al., 2018). In accordance with this, the social identity model of behavior change describes key actions that group facilitators can apply to encourage the formation of SSI amongst intervention recipients (Tarrant et al., 2020). Purposely targeting social identity processes may be essential in those group contexts which rely on a positive group dynamic for the smooth functioning of the intervention (e.g., peer support groups; psychotherapy groups; shared activity groups) (Tarrant et al., 2020).

## Shared Social Identity and Singing Groups

It has been argued that joint music-making activities are uniquely placed to promote SSI through their use of synchronous activity that involves shared attention and achievement (Launay et al., 2013; Weinstein et al., 2016; World Health Organisation, 2019). Group-based singing allows for social interaction that is inclusive of the whole group and not bound by turn-taking as with other social interaction (Kreutz, 2014; Weinstein et al., 2016). In accordance with this, some research has found greater self-reported social connectedness, social identification and qualitative reports of a sense of belonging among those participating in group-based singing interventions (Clift and Hancox, 2001; Joseph and Southcott, 2015; Pearce et al., 2015; Weinstein et al., 2016; Dingle et al., 2020). A number of studies have also evidenced this social bonding effect in singing groups by showing elevation of endorphins linked to positive affect and affiliative behaviors (Kreutz, 2014; Weinstein et al., 2016). However, other research has indicated that it may not be singing, but instead the experience of group-based activities in general and greater time spent together that predict greater connection with the group (Pearce et al., 2016; Bullack et al., 2018). Building upon this research, the current study measures and examines the progression of SSI within a group-based singing intervention as a key mechanism through which it might cultivate positive outcomes.

The Singing for People with Aphasia (SPA) trial is a pilot randomized controlled trial (RCT) of a group-based singing intervention aimed at improving psychosocial outcomes in people with stroke-related aphasia (Tarrant et al., 2018, 2021). Beyond difficulties in language function, many people with post-stroke aphasia report experiences of social exclusion and reduced levels of social activity (Cruice et al., 2006; Parr, 2007; Vickers, 2010). The SPA intervention was informed by the social identity approach to health (Haslam et al., 2018) and the social identity model of behavior change (Tarrant et al., 2020), and was designed to encourage the formation of SSI among the recipients (Tarrant et al., 2018). Alongside singing and music-making, other activities were built in to the intervention to encourage this process, including group-level feedback and encouragement from facilitators, identification of similarities between group members (e.g., recognizing shared interests in music and singing), group goal setting, inclusive language, and also refreshment breaks to encourage member social interaction and personal disclosure (Tarrant et al., 2016). The trial process evaluation included an assessment of the group processes that were active during the intervention sessions—and specifically the extent to which group members exhibited a sense of SSI.

Within group-based singing and other group-based interventions for health, self-report measures are most commonly used to assess SSI (or similar), usually at a single time-point, or two time-points (e.g., before and after delivery of the intervention) (Pearce et al., 2015; Weinstein et al., 2016; Steffens et al., 2019). The administration of self-report measures was deemed to be unacceptably burdensome to SPA trial participants, many of whom had severe language impairments as a result of their aphasia. The SPA trial therefore employed an observational approach that involved the video-recording of the group sessions for later analysis by independent coders. As well as alleviating concerns about participant burden, video-recording of group sessions allowed for assessment of the progression of SSI across the course of the intervention [and could also capture potentially important fluctuations in it (Dunton, 2018)]. The assessment of SSI using observational methods is a novel approach in social identity research; and so there is no validated tool specifically targeting this construct in this way. The study therefore adopted a measure previously used within group therapy contexts that assesses the degree of “cohesiveness” that group members exhibit. Group cohesiveness is closely associated with SSI, both theoretically and empirically and allows for the observation of behaviors indicative of a sense of connection between group members (Hornsey et al., 2007).

## Materials and Methods

### SPA Pilot Feasibility RCT

Full methodology for the current pilot RCT has been reported elsewhere (Tarrant et al., 2018, 2021). Individuals eligible for participation in the trial were: those with post-stroke aphasia; 18+ years of age; and had completed any post-stroke speech and language rehabilitation. Individuals currently participating in singing groups or other lifestyle interventions were excluded from the trial. Forty-one participants were recruited to the RCT. These individuals were randomized 1:1 to receive either the SPA intervention along with a resource pack in aphasia-friendly format, which provided information on living with aphasia and available local community services (*N* = 20), or to the control arm (*N* = 21) in which the participants received the resource pack only. Singing groups were run in three locations across the South West of England—Group A (*N* = 7), Group B (*N* = 7), and Group C (*N* = 6). Group sessions were facilitated by an expert community music leader and assisted by a person with aphasia. The intervention comprised ten weekly sessions, with each session lasting 90 min. There was flexibility to the structure and content of the sessions (e.g., groups were allowed to choose which songs to sing), but each session included: a settling-in phase, a singing warm-up, and a main singing phase which was punctuated by a break lasting around 20–30 min. Each group established a group goal that involved working toward a final activity (performance for family/friends or development of a “playlist”).

### Assessment of Shared Social Identity

The first nine sessions of the intervention were video-recorded (set-up and operated by a researcher) for all three groups. The tenth session was recorded for only one group—Group B—because Groups A and C both chose to do a public performance for their 10th session and we did not have consent to film this. For each group, the 90-minute recording was divided into nine, 10-minute segments, giving approximately 81 segments per group (90 for Group B)^1^ (World Health Organisation, 2019). These segments were randomized in order to blind the independent coders to the segment’s position within the programme, therefore mitigating the risk of biased ratings.

A standard operating procedure was developed for the coding of the video data from the singing sessions. SSI was assessed using the single item measure of global cohesiveness from Budman et al. (1987)’s Harvard Community Health Plan Group Cohesion (HCHP-GCS) scale. Using this scale, each session segment was rated on the degree to which the group *as a whole* appeared cohesive vs. fragmented. Ratings were made on an 11-point bipolar scale, ranging from −5 (very strong fragmentation) to + 5 (very strong cohesion). A score of zero indicated that there was no evidence of either fragmentation or cohesion (i.e., that the construct was not present). Two independent coders were trained (∼3h of training) to code video segments using the standard operating procedure. The training taught coders to consider the extent to which the group members collectively displayed a sense of “groupiness,” as reflected in (high) levels of involvement in the session activities, both while singing (e.g., singing together) and also during the planned session breaks (e.g., engagement in group conversations). Training was iterative in that an initial sample of coding was compared against ratings provided by members of the research team. If ratings departed by more than two points on the scale, coders performed another round of training and the standard operating procedure was revised where necessary. This process was followed until consensus between raters was established (this happened after three rounds of rating). One independent rater coded all video segments (all three intervention groups), and the second coder rated all segments from a single group; an inter-rater agreement score was calculated for this group.

### Statistical Analysis

After completion of the coding, the video segments were re-ordered into their original sequence for analysis. Degree of coder agreement was assessed with a Pearson bivariate correlation between each coder’s average cohesiveness session score. Cohesiveness scores for each session were calculated for each group (i.e., the nine Group A segment scores for week 1 were averaged to create an overall score for that particular session of the programme for that group). Change in global cohesiveness between week one and the final recorded session, for each singing group, was assessed using paired sample *t*-tests. All analyses were completed using IBM SPSS Statistics 25.

## Results

Table 1 presents participant characteristics for each of the three singing groups.

**TABLE 1:** Participant demographics within each singing group.

	Group A	Group B	Group C
	*N* = 7	*N* = 7	*N* = 6
Gender: *N* females	2	2	4
Age (years): *M* (SD)	64 (15.15)	62 (9.49)	71 (11.01)
*N* attending ≥ 80% sessions	5	5	4
*N* mild aphasia^1^	4	5	5
Time since stroke (years): *M* (SD)	5 (4.97)	5 (3.93)	3 (1.76)
*N* carers/guests in attendance^2^	2	3	3

*^1^Other participants having “moderate or severe” aphasia. The very short version of the Minnesota Aphasia Test (Powell et al., 1980) was administered at baseline. Participant responses were reviewed by CC, an aphasiologist, who calculated severity and type of aphasia. ^2^Participants’ carers/guests did not attend every session and did not always join in the singing/group activity. The facilitator was present at each session and the singing champion was present at almost all sessions.*

### Cohesiveness

**FIGURE 1:**
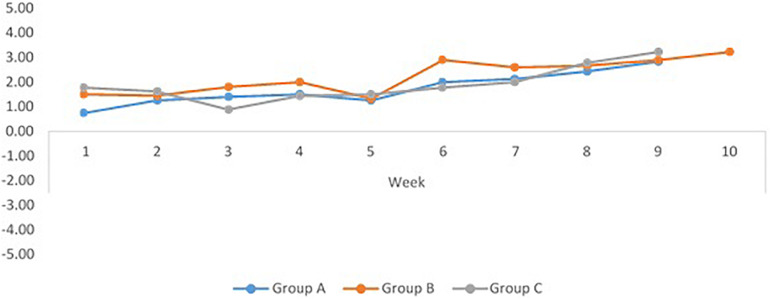
Progression of global cohesiveness across the intervention programme (weeks 1–10), for each singing group (negative scores indicate fragmentation; positive scores indicate cohesiveness).

There was a high level of agreement in cohesiveness ratings between the independent coders *r*(9) = 0.85, *p* < 0.005. Patterns of group cohesiveness were consistent across each of the three cohorts. At the beginning of the intervention, each of the three groups reported relatively low levels of cohesiveness (with no scores < 0, indicating no fragmentation) and there was a clear upward trend in cohesiveness across the intervention (Figure 1). Cohesiveness was reliably higher at the end of the intervention compared to the beginning, for all three groups (Table 2).

**TABLE 2:** Cohesiveness at the beginning and end of the intervention, by group.

	First		Final		Comparison		
	Session		Session		between sessions		
Cohort	M	SD	M	SD	t	df	*p*
Group A (*N* = 6*)	0.67	0.82	2.83	0.41	−5.40	5	0.003
Group B (*N* = 9)	1.56	0.53	3.22	0.44	−7.07	8	<0.001
Group C (*N* = 9)	1.78	0.83	3.22	0.44	−4.27	8	0.003

*Six segments only: See footnote 1.

## Discussion

While groups are now a common mode of delivery for a wide range of behavior change and health interventions—including arts and music-based—understanding of the social identity processes that may shape an intervention’s impact is limited and rarely targeted in formal trial evaluations (Steffens et al., 2019). The current paper therefore examines social identity within the SPA intervention, designed specifically to encourage the development of SSI among intervention recipients (Tarrant et al., 2016). SSI, usually captured through retrospective self-reports, was instead assessed using an observational proxy: group cohesiveness (Budman et al., 1987). The analysis indicated a clear upward trend in group cohesiveness (indicative of SSI formation) across the intervention group sessions, with the final session evidencing reliably higher levels of cohesiveness than the initial session. These effects were found to be consistent across the three groups investigated, suggesting that the participants internalized the group intervention in similar ways. Interviews with intervention facilitators and participants who took part in the research support this conclusion and indicated that the SPA intervention promoted a strong sense of SSI between recipients [reported elsewhere (Tarrant et al., 2021)].

These findings give greater confidence both that this novel approach to investigating SSI warrants further investigation as an indicator of the construct (Hornsey et al., 2007), and also that the intervention elicited the intended social identity processes. Methodologically, the observational approach has allowed for an analysis of the progression of SSI in a way that would not have been possible with other more common, retrospective, self-report approaches, and in a way that did not burden the research participants. Extending this point, the high levels of inter-rater reliability achieved here demonstrate the potential feasibility of employing observational methods to capture group phenomena and opens up the possibility that this approach could similarly be employed to measure other group processes that contribute to (and flow from) SSI in intervention settings, such as social support, positive social influence, and group efficacy (Haslam et al., 2018).

An interesting question arises concerning the potential contribution of different intervention activities to the development of shared social identity. In the current analysis, our focus has been on shared social identity at the level of the session as a whole (i.e., comparing session cohesiveness scores across the intervention programme). It is possible that some session elements may have more strongly contributed to the development of shared social identity than others—for example, the more “informal” elements that allow for the natural formation of social connections (e.g., unstructured conversation during arrival at/departure from the session; mid-session breaks). Although we did not code individual participant actions that may be expected to contribute to the development of cohesiveness in such elements, the current approach to analysis, which involved the coding of pre-determined timed segments, can provide a basis for future research where a more targeted analysis is demanded by the research questions (e.g., coding singing vs. non-segments elements).

### Study Limitations

The aim of the current study was to document the progression of SSI across the course of a group-based singing intervention. The findings do not allow for any inferences to be made regarding the consequences of developing SSI for the intervention outcomes. A priority for a future, definitive trial of the SPA intervention will be to test the effectiveness of the intervention on health outcomes; the process evaluation for that trial will formally test whether these outcomes are causally related to SSI developed within the context of the intervention group (and indeed to any fluctuations in SSI). The current study outlines a methodology that can support such an assessment of trial processes.

Despite its clear advantage in terms of supporting the detailed examination of social identity processes, the observational approach adopted here is resource intensive in terms of the time required to code the data. It is also possible that the mere presence of a video camera and operator during group sessions may be experienced by some participants as intrusive, and this itself may affect the group’s behavior—although we point out that there was no evidence of this in the current trial. However, repeated administration of self-report measures of SSI and other group processes may also be intrusive, and likely fatiguing for some, especially those living with chronic health conditions. With careful management of the group’s expectations, any potential negative impacts of video recording may be minimized.

Finally, while the current analysis indicated a clear upward trend in group cohesiveness, or SSI, it is not possible to say here what a “desirable” level of this construct might be (i.e., there is no clinically significant value assigned to the social identity construct). Thus, while there was a reliable difference between scores at the beginning and end of the SPA intervention (and no segment scores that indicated group *fragmentation*), the precise *meaning* of this difference beyond it being *different* is unclear. This is not an issue unique to this methodology: the authors are not aware of any research which has sought to determine the meaning of individual scale points on (self-report) measures more commonly employed to assess SSI. It would be of obvious benefit to future process evaluations of group-based interventions (and social identity research more generally) to be able to determine the level of SSI that is necessary to shape the desired intervention outcomes.

## Conclusion

This study employed a systematic observational method to investigate a group process shown previously to be a key determinant of health: SSI. The analysis revealed a steady increase in a proxy measure of this construct (group cohesiveness) across the course of the 10-week group-based singing intervention. Beyond allowing for the testing of fidelity of this kind of singing intervention, the study has helped establish a methodology by which SSI can be assessed directly, *in situ*. As well providing a non-burdensome alternative to repeated self-report measurements, this methodology may allow for the mapping of behavioral exemplars and other key group processes that flow from SSI, and can thereby enhance future process evaluations of group-based interventions.

## Data Availability Statement

The datasets presented in this article are not readily available because participants did not consent for datasets to be stored or accessed outside of the research team. Requests to access the datasets should be directed to MT, m.tarrant@exeter.ac.uk.

## Ethics Statement

The trial was reviewed and approved by the Health Research Authority (HRA) and NHS National Research Ethics Service and Research, *via* the Southwest—Frenchay Research Ethics Committee (17/SW/0060). The patients/participants provided their written informed consent to participate in this study.

## Author Contributions

MT led the project team and was principal investigator. RL led the completion of the manuscript. MC was trial researcher. SD led the main process evaluation of the SPA trial. SS served as independent coder of session segments. AS was trial researcher and supported trial closure. RC was trial manager and researcher and supported all aspects of the trial. All authors reviewed the article for publication.

## Conflict of Interest

All authors report the Stroke Association funding for the work under consideration but no other conflicts of interest.

## Footnotes

1 There was some minor variation in total number of segments per session across the three groups due to video camera failure (e.g., week nine of Group A, when there were only six segments) or due to the session finishing slightly early. One session overran, creating more than nine segments (week two of Group B had 11 segments).
